# Supplementary material for: Macrophage-like Blood Cells Are Involved in Inter-Tissue Communication to Activate JAK/STAT Signaling, Inducing Antitumor Turandot Proteins in Drosophila Fat Body via the TNF-JNK Pathway
Source: Int J Mol Sci. 2024 Dec 6;25(23):13110. doi: 10.3390/ijms252313110 (PMC11641801; doi:10.3390/ijms252313110)
Supplement: Supplementary file 1 [file ijms-25-13110-s001.zip › ijms-3270687-supplementary.pdf]

Supplementary Figure Legends

Fig. S1. Anti-MMP1 immunostaining of the normal circulating hemocytes transplanted into control and mxc^{mbn1} larvae.

(a, b) Fluorescence images of circulating hemocytes from normal larvae at mature third instar stage in normal (*w/Y;He>GFP*) (a) and mxc^{mbn1} (b) larvae (mxc^{mbn1}*/Y;He>GFP*). Scale bar: 10 µm; blue, DNA; green, transplanted hemocytes; red, anti-MMP1 immunostaining signal. (c) The relative fluorescence intensity of anti-MMP1 immunostaining. The fluorescence intensity of each transplanted hemocyte was quantified and displayed on the y-axis relative to the fluorescence intensity of the normal control set at 1. x-axis from left to right: normal control (*w/Y;He>GFP*) (n = 139), mxc^{mbn1} (mxc^{mbn1}*/Y;He>GFP*) (n = 32), The average fluorescence intensity is shown as a red line. (Welch's *t* test, *p<0.05). Error bars indicate the SEM.

Fig. S2. Enhancement of the LG tumor's growth in mxc^{mbn1} larvae harboring *wgn*- or *grnd*-depleted circulating hemocytes.

(a-d) DAPI-stained images of LGs from mature larvae at late third instar stage. (a) normal control (*w/Y*), (b) mxc^{mbn1} larvae expressing hemocyte-specific dsRNA against *GFP* mRNA (mxc^{mbn1}*/Y;He>GFPRNAi*), (c) mxc^{mbn1} harboring hemocyte-specific depletion of *wgn* (mxc^{mbn1}*/Y;He>wgnRNAi*), (d) mxc^{mbn1} harboring hemocyte-specific depletion of *grd* (mxc^{mbn1}*/Y;He>grndRNAi*^{2}). Scale bar is 100 µm. (e) Quantification of the LG sizes of mature larvae at third instar stage. From left to right: normal control larvae (*w*/Y), mxc^{mbn1} mutant larvae (mxc^{mbn1}*/Y;He>GFPRNAi*), mxc^{mbn1} with LG sizes of the larvae with hemocyte-specific *wgn* depletion (mxc^{mbn1}*/Y; He>wgnRNAi*), thew mutant larvae with the *grnd* depletion (mxc^{mbn1}*/Y;He>grndRNAi*) (one-way ANOVA with Bonferrer) (One-way ANOVA with Bonferroni correction, *p<0.05, ***p<0.001,****p<0.001, n = 20 (*w/Y*), n = 20 (mxc^{mbn1}*/Y;He>GFPRNAi*), n = 19 (mxc^{mbn1}*/Y;He>wgnRNAi*), n = 42 (mxc^{mbn1}*/Y;He>grndRNAi*). The red line indicates the mean LG size, and the error bars indicate the SEM.

Sup.Fig.S1

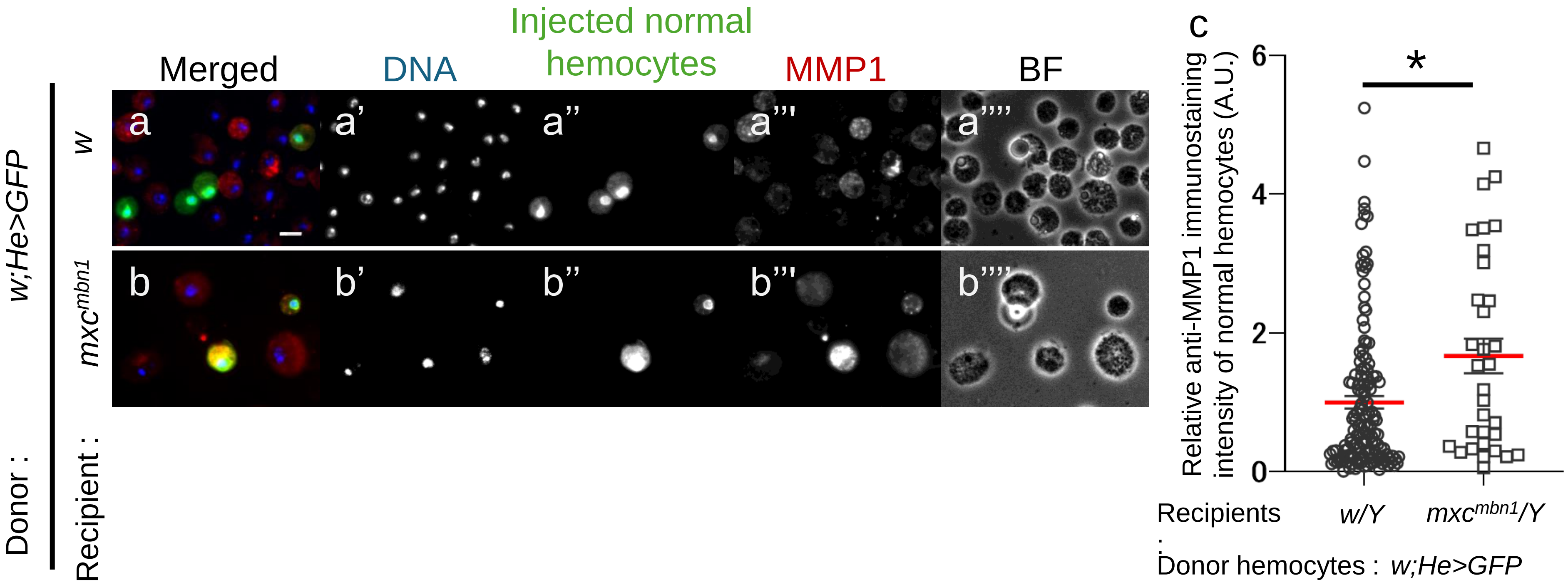

Merged
DNA
Injected normal hemocytes
MMP1
BF
a
a'
a''
a'''
a''''
b
b'
b''
b'''
b''''
w
mxc^mbn1
w;He>GFP
Donor :
Recipient :
c
*
6
4
2
0
Relative anti-MMP1 immunostaining intensity of normal hemocytes (A.U.)
Recipients :
w/Y
mxc^mbn1/Y
Donor hemocytes : w;He>GFP

Sup.Fig.S2

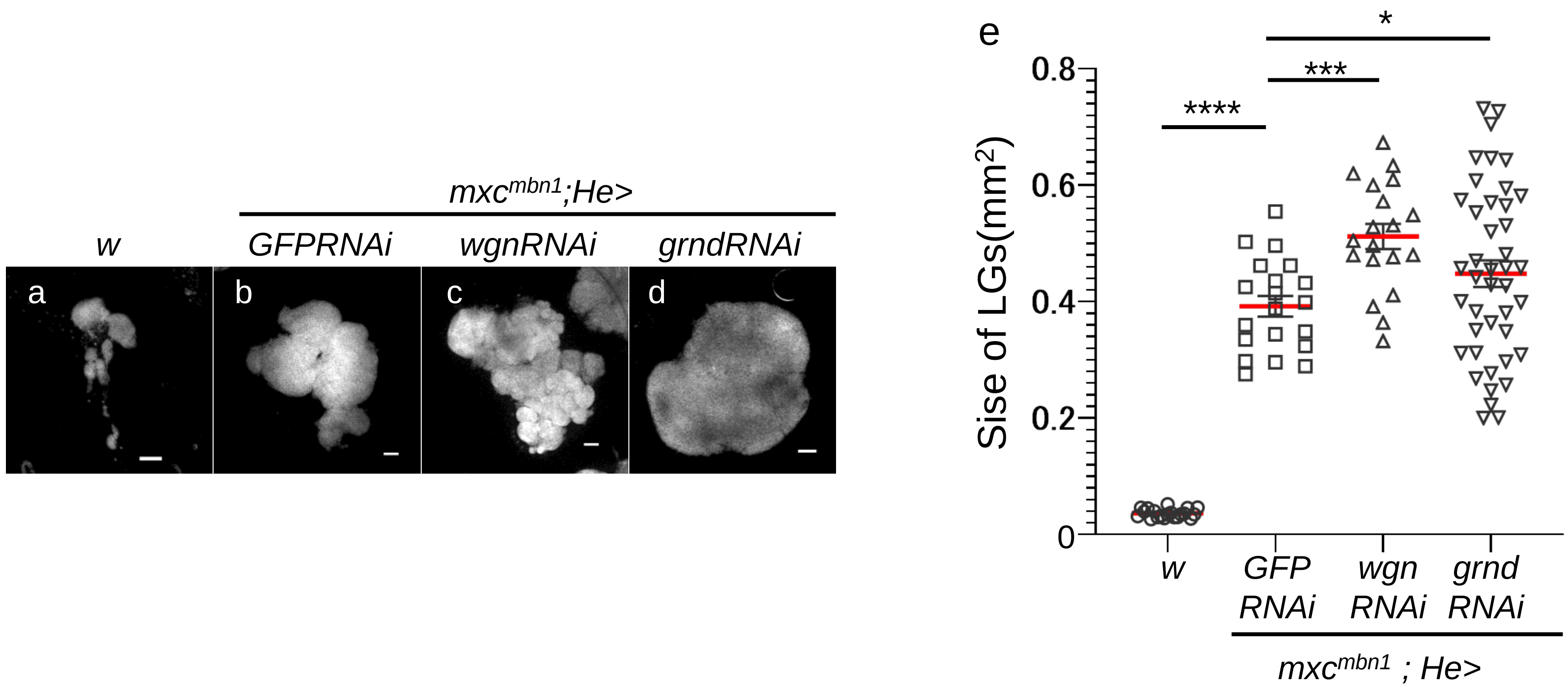
mxcmbn1;He>
w
GFPRNAi
wgnRNAi
grndRNAi
a
b
c
d
e
Sise of LGs(mm2)
0.8
0.6
0.4
0.2
0
****
***
*
w
GFP RNAi
wgn RNAi
grnd RNAi
mxcmbn1 ; He>
